# Intestinal Barrier Function and Performance of Broiler Chickens Fed Additional Arginine, Combination of Arginine and Glutamine or an Amino Acid-Based Solution

**DOI:** 10.3390/ani11082416

**Published:** 2021-08-17

**Authors:** Reza Barekatain, Tristan Chalvon-Demersay, Clive McLaughlan, William Lambert

**Affiliations:** 1 South Australian Research and Development Institute, Roseworthy Campus, University of Adelaide, Roseworthy, SA 5371, Australia; Clive.McLaughlan@sa.gov.au; 2 METEX NOOVISTAGO, 32 Rue Guersant, 75017 Paris, France; Tristan.Chalvon-Demersay@metex-noovistago.com (T.C.-D.); William.Lambert@metex-noovistago.com (W.L.)

**Keywords:** intestinal permeability, dexamethasone, inflammation, barrier function, tight junction proteins, gene expression

## Abstract

**Simple Summary:**

There is growing evidence that amino acids can influence intestinal barrier function and inflammation in broiler chickens. Arginine, glutamine, and threonine are regarded as functional amino acids that can help restore gut integrity-related issues under enteric or stress-related conditions. The present study aimed to investigate different combination of arginine, glutamine, and threonine plus a grape extract specifically tailored to improve performance and intestinal functions of broilers. The results showed that tested amino acids were able to improve feed conversion ratio of broilers while alleviating the intestinal inflammation caused by administration of synthetic glucocorticoid through different pathways. There was also indication of changes in intestinal permeability by tested amino acids. The mechanistic understanding of different amino acids and their combinations presents opportunity to optimize intestinal barrier function particularly under stress related conditions.

**Abstract:**

Two experiments were conducted to investigate the effect of arginine (Arg); the combination of Arg and glutamine (Gln); as well as an amino acid-based solution (MIX) containing Arg, Gln, threonine (Thr), and grape extract, on performance, intestinal permeability, and expression of selected mechanistic genes. Using 240 male Ross 308 off-sex broiler chickens, four experimental treatments were replicated six times with 10 birds per replicate. The experimental treatments included 5 g/kg Arg, 2.5 g/kg Arg and 2.5 g/kg Gln, and 1 g/kg MIX added to a basal diet as control. In the second study, the four dietary treatments were then given to 24 birds with or without a synthetic glucocorticoid, dexamethasone (DEX), as a gut dysfunction model. Feed conversion ratio was improved by all the supplemented treatments from day 7 to 35 of age (*p* < 0.001). DEX injections increased (*p* < 0.001) the intestinal permeability in all treatments, which tended to be reversed by Arg or MIX. Additional Arg, Arg-Gln, and MIX suppressed (*p* < 0.05) the overexpression of *IL-1β* generated by DEX. Feeding birds with MIX treatment increased (*p* < 0.05) expression of *SGLT-1* and glutathione synthetase. In conclusion, tested amino acid supplements were effective in improving feed efficiency and restraining intestinal inflammation caused by DEX through *IL-1β* pathway.

## 1. Introduction

Amino acids play fundamental roles in the optimum functionality of the intestine with increasing evidence of regulation of intestinal barrier function by concentration of amino acids in the diets [1]. There is evidence for the role of amino acids such as glutamine (Gln), arginine (Arg), and threonine (Thr) having positive effects on controlling permeability, promoting cell proliferation, and stimulating several metabolic pathways such as mTOR [1,2].

As an essential amino acid for chickens, mounting evidence is emerging on various fundamental roles that Arg plays in different metabolic pathways, regulation of intestinal function, and ultimately protein synthesis and performance. For example, the synthesis of nitric oxide and polyamines is dependent on Arg [3]. Production of nitric oxide along with enterocyte migration is crucial for restoration of epithelial continuity [4]. Therefore, Arg can modulate immune response, inflammation, and process of recovery from injury or stress. Recent data with poultry suggest that there is a potential for increasing feed specification for Arg beyond current industry practices to support optimum growth and intestinal functions [5]. Supplementation of Arg can therefore improve the gut barrier function as shown by reducing the ileal permeability measured in Ussing chambers by Zhang, et al. [6]. The specific role of Arg in low protein diets for intestinal barrier function has recently been investigated [7] and showed supplementing additional 5 g/kg Arg positively impacted feed efficiency and some aspects of intestinal permeability and tight junction expression. However, such results need to be confirmed. More importantly, additional Arg may not be economically feasible at such high supplementation level. Therefore, the combination of amino acids or specifically tailored products may be suitable alternatives. Gln and Thr also serve as functional amino acids with specific effects on intestinal permeability and immune function of broiler chickens [8,9]. The combination of these amino acids is hypothesized to influence intestinal barrier function by promoting growth performance, anti-inflammatory effects, and underlying mechanisms associated with such response. 

Polyphenolic compounds including flavonoids are commonly plant-derived compounds known for having antioxidant capacity and protecting different cells from oxidative damage [10]. With growing evidence for some phenolic compounds to improve gut health and performance of broiler chickens, it is hypothesized that inclusion of grape extract containing flavonoids alongside amino acids (as part of an amino acid-based supplement) may further enhance the intestinal barrier function and reduce stress-induced intestinal inflammation. To our knowledge, there is no previous research in which such approach has been tested to investigate the mode of action of different combinations of amino acids or polyphenols in counteracting intestinal inflammation, enteric and stress-related issues in broiler chickens. 

Intestinal barrier dysfunction can be induced by various physiological or nutritional factors [11]. One such model is through the use of dexamethasone (DEX), a synthetic glucocorticoid (GC) that causes increased intestinal permeability, differential expression of tight junction proteins, and increased inflammation [12]. As the use of GC can increase gluconeogenesis and therefore accelerate catabolism of amino acids [12], this model may be useful to investigate possible counteracting and mechanistic roles of amino acids at least at the intestinal level.

Thus, the current project was conducted to investigate the effects of additional Arg; a combination of Arg and Gln; or an amino acid-based solution containing Arg, Gln, Thr, and grape extract on performance, intestinal barrier function, and mechanistic genes involved in gut barrier function, inflammation, protein synthesis, and apoptosis using DEX as a gut barrier dysfunction model. 

## 2. Materials and Methods

Animal Ethics Committee of Primary Industries and Regions approved all procedures of this study (7/19). 

### 2.1. Experimental Design and Diets

The study involved a performance experiment to assess the effect of experimental treatments on growth performance of broiler chickens and a challenge experiment to investigate intestinal barrier function, intestinal permeability, and mechanistic gene expression assays under normal and stress-stimulated conditions. As shown in Table 1. basal grower and finisher diets were formulated to meet or exceed the nutrient specifications of Ross 308 (2019) after analyzing ingredients for amino acids and proximate analysis using NIR. Grower diets were fed from day 7 to 21 followed by a finisher until day 35 of age. Five g/kg sand was incorporated in the basal diets to which tested amino acids were substituted. Four experimental diets were constructed by including basal diet as control (CON), Arg (5 g/kg), a combination of Arg (2.5 g/kg) and Gln (2.5 g/kg), and an amino acid-based product (MIX). The MIX contained Arg, Gln, and Thr plus a grape extract (5%) was separately prepared, mixed, and supplemented at 1 g/kg of diet to compose the fourth treatment. The inclusion rate of MIX (1 g/kg) was based on an unpublished dose-response study conducted by METEX NOOVISTAGO [13]. The grape extract used had a minimum of 70% polyphenols content. All diets were mixed and pelleted using a cold-press pelleting machine at feedmill facility of the South Australian Research and Development Institute (SARDI), Roseworthy Campus. All experimental diets were analyzed for amino acid composition as shown in Table S1 (Supplementary Materials). 

A total of 336 male off-sex day-old Ross 308 chickens were obtained from Aviagen Hatchery (Goulburn, New South Wales and were transferred to SARDI Poultry Facilities at Roseworthy, South Australia The chicks were vaccinated for infectious bronchitis and Newcastle disease viruses at hatchery. Upon arrival, 240 birds were placed in 24 raised-floor pens each accommodating 10 birds. The remaining 96 birds were given eight additional pens for later use in the challenge part of the study. All birds were given the same commercial starter diet until day 7 of age. Each of the four diets was given to six pens for the performance assessment and two additional pens for the challenge study. Wood shavings were used as bedding materials. Feed and water were provided ad libitum throughout the study. Temperature was maintained around 32 °C in the first 2 days and then gradually decreased until a constant temperature of 23 °C was reached on day 21 of age. Birds were given a lighting program of 16 h light and 8 h dark after day 2 of age. In the first 2 days of age, birds were given 24 h light. 

On days 0, 7, 21, and 35, birds were weighed and feed intake recorded for each pen. Feed conversion ratio was then calculated and corrected for mortality if it occurred. 

### 2.2. Challenge Trial

On day 7, the remaining 96 birds were also assigned to four experimental treatments and were kept in raised-floor pens until day 13 when birds were transferred to 96 individual cages. The environmental conditions including temperature and lighting programs were the same as the birds on floor pens reared in a separate shed. Using a 2 × 4 factorial arrangement of treatments, each diet was replicated 24 times by assigning diets to 24 birds, half of which were injected intramuscularly in breast with DEX on day 14, 16, and 20 of age at 0.5 mg/kg body weight (BW) to induce gut barrier dysfunction similar to previous studies [7,12]. The remaining birds were given sham injections of sterile saline solution (0.9%) on the same days. DEX solution was prepared following the procedure described by Wideman Jr and Pevzner [14]. Briefly, a stock solution was prepared by dissolving DEX (Sigma-Aldrich, St. Louis, MO, US in 95% ethanol. The working solution was prepared fresh on each day of injections by making a final solution of 0.5 mg/mL of DEX in sterile saline solution (0.9%). Feed intake of each bird was recorded for the entire 1-week study in the cages. The weight of the birds was recorded at the beginning, on the days of injections, and at the end of the 1-week study.

### 2.3. Intestinal Permeability Assay and Sampling 

Fluorescein isothiocyanate-dextran (FITC-d; 4000 Da; Sigma-Aldrich, St. Louis, MO, US) was used to assess intestinal permeability following the procedure described by Vicuña, et al. [15] with some modifications. Accordingly, on day 21, each bird was orally gavaged with 4.16 mg/kg BW of FITC-d dissolved in water. A working solution of FITC-d was prepared by dissolving 4.16 mg FITC-d per 1.5 mL of Milli-Q water. To obtain the right volume/dosage based on BW, each bird was weighed prior to administering the solution and the volume required for each bird was calculated using an already prepared Excel (Microsoft, Washington, DC, US) spreadsheet template. By this calculation, we made sure that each bird received at least 1 mL of solution while still receiving a precise 4.16 mg/kg BW. After 150 min, a blood sample was taken from the jugular vein from live birds. Birds had access to feed and water during the 150 min procedure. Blood samples were left at room temperature and then centrifuged at 1500× *g* for 15 min. Triplicate measurements were conducted on each sample and standard solutions. A previously stored serum sample from birds without FITC-d was utilized as a blank. A Synergy MX plate reader (Biotek Instruments, Bedfordshire, UK) was used to measure the absorbance at excitation of 485 and emission of 530 nm. 

On the same day, all birds were euthanized by cervical dislocation. After weighing, each bird was dissected and weights of liver, spleen, and bursa were recorded. A sample of ileal tissue was collected from mid-ileum and subsequently rinsed with PBS and snap frozen in liquid nitrogen. All ileal tissues were then stored in −80 °C until used for analysis. 

### 2.4. Gene Expression Assays

RNA extraction process and PCR assays were conducted largely similar to a previous study [16]. Six samples from each treatment were included in the gene expression assays. A RNeasy Plus Universal Mini Kit (Qiagen, Hilden, Germany) was used to isolate total RNA from ileal tissues after homogenization of approximately 100 mg of each tissue sample in Qiazol (Qiagen, Hilden, Germany). Concentration of RNA was measured using a Thermo Scientific™ NanoDrop 2000. The integrity of RNA samples was determined using a Tape Station (Agilent Technologies 2200, Waldbronn, Germany) with samples revealing RIN numbers above 9. All RNA samples were diluted to 200 ng/µL using RNA free water. A high-capacity cDNA reverse transcription kit (Thermo Fisher Scientific, Waltham, MA, US) was utilized to synthesis the cDNA from total RNA following the manufacturer’s instruction. The 1:20 diluted cDNA in 10 mM Tris buffer was used in all PCR assays. Primers of each gene including reference ones are given in Table 2. All PCR assays were conducted using a standard curve method and Power SYBR™ Green PCR Master Mix (Life Technologies Ltd., Warrington, UK). Values were from triplicate measurements using a QuantStudio™ 6 Flex (Thermo Fisher Scientific, Waltham, MA, US). A 1:4 pooled cDNA sample of the same ileal tissues was used to construct the standard reactions in each assay. All quantified values of the assayed genes were divided by the quantified values of *TBP* as the reference gene because this gene was found to be most stable. Therefore, relative values were used for statistical analysis. The RT-PCR run method included an initial 10 min denaturation at 95 °C and PCR conditions were 95 °C for 15 s, 60 °C for 20 s, and 72 °C for 40 s. A melting curve stage was also added. 

### 2.5. Statistical Analysis

Data of the experiment were analyzed using General Linear Model of SAS 9.4 (SAS Institute Inc., Cary, NC, US). For performance parameters, data were subjected to a one-way ANOVA. For the challenge part of the study, all data were subjected to two-way ANOVA to assess the effect of diet, DEX challenge, and their interaction. Data were checked for normal distribution and they were normally distributed. Fisher’s least square differences test was used to separate the differences between means when a significant difference for main effects or interaction was detected. The level of significance was set at *p* < 0.05 while tendency was considered for 0.05 ≤ *p* ≤ 0.10. 

## 3. Results

### 3.1. Growth Performance

Table 3 and Table 4 show the growth performance data of birds from day 7 to 35 of age. Only 3 birds died (1.25% mortality) throughout the experiment 1 with no significant association with dietary treatments. Body weight, body weight gain, and feed intake of birds were not affected by the experimental diets at any stage of the experiment. However, FCR was improved by supplementing Arg, Arg, and Gln or MIX from day 7 to 21 (*p* < 0.01) and when assessed from day 7 to 35 (*p* < 0.001). 

The individual 1-week performance of birds in metabolism cages from day 14 to 21 was not affected by the diets (Table 5). During the same time, there was a profound effect of DEX to reduce (*p* < 0.0001) body weight gain, feed intake, and increase (*p* < 0.0001) FCR.

### 3.2. Relative Weights of Liver, Bursa and Spleen

As shown in Table 6, there was no interaction between DEX and diet for immune organ weights. Supplementation of tested amino acids and MIX independently reduced (*p* < 0.001) the relative weight of liver. DEX increased (*p* < 0.0001) the liver weight compared with sham-injected birds. Relative weights of bursa and spleen were not affected by dietary treatments while they were markedly reduced by DEX (*p* < 0.0001). 

### 3.3. Intestinal Permeability

**Figure 1:**
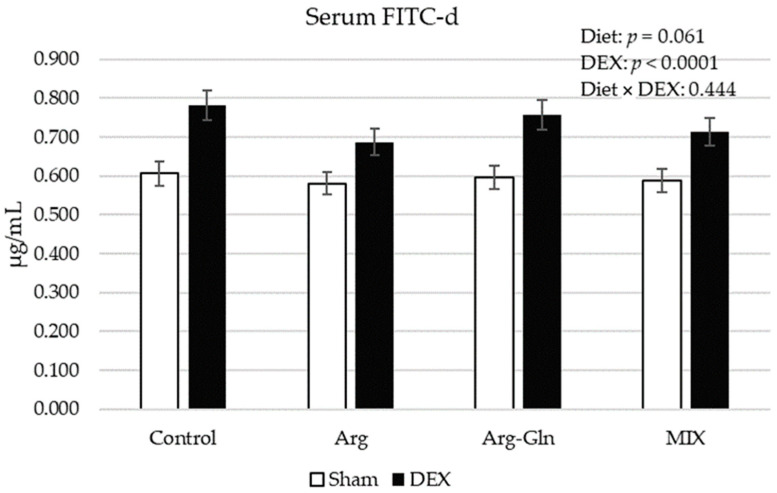
Serum concentration of FITC-d on day 21 of age. Error bars represent pooled standard error. FITC-d: Fluorescein isothiocyanate-dextran; MIX: A supplement that contained Arg, Gln, Thr, and grape extract; Diet × DEX: interaction of diet and DEX; DEX: Injected with dexamethasone; Sham: Injected with saline solution.

Figure 1 shows the FITC-d concentration in serum of broilers on day 21 of age. DEX challenge increased (*p* < 0.001) the concentration of FITC-d. Dietary treatments tended (*p* = 0.06) to decrease the level of FITC-d with the lowest level values observed for Arg-fed birds. 

### 3.4. Ileal Gene Expression Assays

Relative mRNA expression data of tight junction proteins in ileum of broilers are given in Table 7. Expression of *Claudin 1* was not affected by either diets or DEX injections. *Claudin 3* was upregulated by DEX (*p* < 0.001) while it remained unaffected by experimental diets. DEX independently increased Zonula occludens 2 (*ZO2*) (*p* < 0.001) and tended to decrease (*p* = 0.078) Zonula occludens (*ZO1*) expression compared with sham-injected birds with no diet effect. 

**Figure 2:**
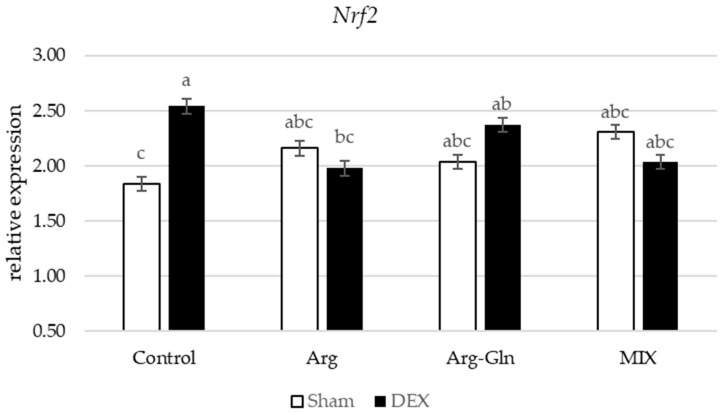
Interaction between diet and dexamethasone for relative mRNA expression of Nuclear factor-erythroid factor 2-related factor 2 (*Nrf2*). ^abc^ Bars not sharing the same letter differ significantly (*p* < 0.05). Error bars represent pooled standard error. DEX: Injected with dexamethasone; Sham: Injected with saline solution.

**Figure 3:**
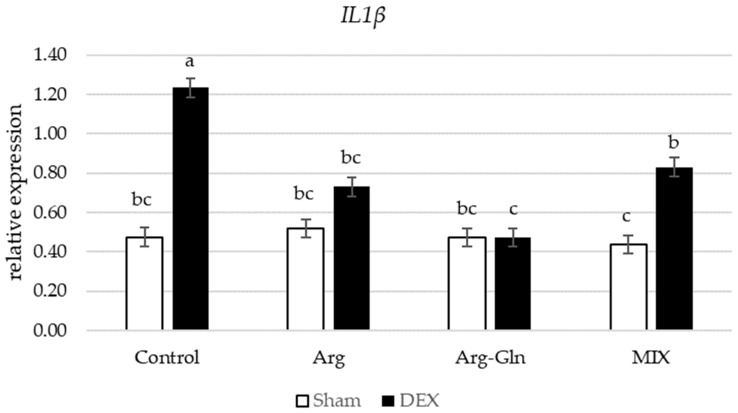
Interaction between diet and dexamethasone for relative mRNA expression of Interleukin-1β (*IL1β*). ^abc^ Bars not sharing the same letter differ significantly (*p* < 0.05). Error bars represent pooled standard error. DEX: Injected with dexamethasone; Sham: Injected with saline solution.

Amongst all gene’s assays in this study, there was an interaction between DEX and diets for Nuclear factor-erythroid factor 2-related factor 2 (*Nrf2*) (*p* < 0.05) and Interleukin-1β (*IL1β*) (*p* < 0.05) (Figure 2 and Figure 3). Supplementation of additional Arg, Arg-Gln, and MIX suppressed the overexpression of *Nrf2* by DEX observed for CON group of birds. For *IL1β*, in sham-injected birds, there was no difference between birds fed four dietary treatments while in DEX-injected birds, supplementation of all tested amino acids and MIX reduced the expression of *IL1β*.

Expression of Mucin-2 (*MUC2*) and Interleukin-6 (*IL6*) was not affected by either DEX or dietary treatments. With no effect of experimental treatments, DEX independently upregulated gene expression of Interferon gamma (*IFNy*) (*p* < 0.05) and Interleukin-10 (*IL10*) (*p* < 0.001). DEX also tended (*p* = 0.092) to increase mRNA expression of Nuclear factor kappa B (*NFκB*) in all birds (Table 8). 

Gene expression of Peptide transporter 1 (*Pept1*), Heat-shock protein 70 (*HSP70*), Mammalian target of rapamycin (*mTOR*), B-cell lymphoma 2 (*Bcl2*), and Ribosomal protein S6 kinase beta-1 (*RPS6KB1*) remained unaffected by either DEX or dietary treatments (Table 9). DEX did not change the sodium glucose cotransporter-1 (SGLT1) expression in the ileum but MIX increased (*p* < 0.05) it compared with other three treatments. The level of mRNA for glutathione synthetase (*GSS*) was independently increased (*p* < 0.05) in birds fed a diet completed with MIX compared with CON and Arg-Gln treatments as well as by DEX challenge (*p* < 0.05). Relative gene expression of *Caspase-3* tended (*p* = 0.069) to upregulate by DEX challenge with no effect associated with diets. Feeding birds with a MIX diet increased (*p* < 0.05) the expression of gene encoding Regulatory-associated protein of mTOR (*Raptor*) only compared with birds fed Arg-Gln treatment. 

## 4. Discussion

The growth performance results of the current project exceeded the Ross 308 standards for body weight on day 35 [23]. In line with previous research [7], additional Arg improved FCR at any stage of the study except for the finisher phase. It appears that for optimal feed efficiency of broilers fed reduced protein diets, a higher concentration of Arg than the current industry recommendations may be required. Noteworthy, a combination of Arg and Gln was equally effective in improving the FCR. This result may be related to the presence of corn in the diets compared with previous studies [7,24] conducted with wheat-based diets in which Gln failed to improve growth performance of the birds. Broilers fed corn-based diets are more conducive to Gln supplementation due to lower concentration of Gln in corn relative to wheat particularly when reduced protein diets are used [25]. The positive growth performance by feeding a combination of Gln and Arg may also be explained by their stimulatory effects on gut development (e.g., provision of energy source for enterocytes, synthesis of polyamines), immune response and function, as well as epithelial cells regeneration [2,26].

An amino acid-based solution with the combination of Arg, Gln, Thr, and grape extract with only 1 g/kg dietary inclusion was able to improve performance similar to other treatments. Noteworthy, in the experiment, the objective was not to separate the effect of all individual amino acids or grape extract but rather investigate their combined effects on promoting growth performance and mechanisms surrounding intestinal barrier function of broiler chickens. Therefore, the main part of the study utilized DEX injections as a gut barrier dysfunction model. There was no interaction between DEX and diet for performance-related parameters and permeability, which was expected given the response of birds is markedly higher to DEX than diets. The performance data of experiment 2 were only presented as indicative of the birds’ status. The lack of diet effect on performance parameters of these individual birds is explained by the relatively short stay in cages (one week) as opposed to the group-housed birds on raised floor pens in experiment 1. 

Repeated injections of DEX as a synthetic GC cause strong and wide-ranging effects on intestinal barrier function and inflammation by induing stress-like response when preferentially binding to type II GC receptors [12]. At the systemic level, suppression of immune response was expected as evidenced by atrophy of bursa and spleen observed in the current study, while there were also substantial metabolic consequences as shown by hepatic enlargement in this study that may be the result of metabolic changes in processes such as lipogenesis and steatosis [27]. On the other hand, dietary treatments reduced the weight of liver independently, which may be related to a possible reduction in inflammation or changes in fat accumulation and need further investigation.

Intestinal epithelial cells play crucial roles in maintaining the intestinal barrier to avoid entry of harmful and unwanted bacterial toxins and pathogens while allowing passage of essential nutrients, water, and ions. Intestinal permeability, as the key function of the intestinal barrier, is controlled by tight junction proteins. *Claudins 1* and *3* are among barrier-forming tight-junction proteins and alongside other tight junctions such as zonula occludens from strong crosslinks with F-actin and myosin from the membrane cytoskeleton [28]. Changes in claudin levels and *ZOs* are expected to influence gut barrier function and permeability. In the current study, the ileal tissue was selected to study the gene expression including that of tight junction proteins as it was found in other studies that gut barrier-related genes were comparatively more responsive in the ileum than jejunum in broiler chickens [7,12]. Transcriptional expression of *claudin 1* and *3* as well as *ZO1* and *ZO2* remained unaffected by dietary treatments supplemented with tested amino acids. However, overexpression of *Claudin 3* and *ZO2* in DEX-injected birds was consistent with a previous study [12]. Although increased expression of these tight junctions is not in line with increased permeability observed in DEX-injected birds, it may be related to a compensatory response to restore the intestinal barrier in the challenged birds. This is possible as the tissue sampling was conducted almost 24 h after the last DEX injection in the current experiment. The intestinal epithelial cells and the tight junction proteins are highly dynamic and rapidly replaced [28], therefore, it is possible that within that timeframe these proteins were highly expressed to restore the dysfunctional intestinal barrier.

In the present study, the FITC-d was given proportionate to the BW of the birds while in two other studies in our laboratory the same dosage of FITC-d was given to the birds regardless of their BW [7,12]. Consistently, there was still a significant increase in FITC-d concentration in serum of birds, which may indicate that the damage to the intestinal barrier and tight junction integrity is more critical than BW or dosage of FITC-d for detection of differences in intestinal permeability in broiler chickens. There was a trend for change in intestinal permeability measured by FITC-d associated with diets in which Arg and then MIX tended to decrease the intestinal permeability in a higher proportion than other treatments. This observation concurred with a previous study [7] in the case of Arg. As a functional amino acid, Arg can impact epithelial integrity through its secondary functions including production of nitric oxide and polyamines [29]. However, the response to Arg observed in this study cannot be explained by gene expression of selected tight junction proteins and there may be other mechanisms involved.

Amongst various cytokines, *IL1β*, a proinflammatory cytokine, is involved in the development of intestinal inflammation and depending on its concentration can increase intestinal permeability [30]. This increase in permeability at least in part is reported to be associated with upregulation of myosin light chain kinase [30]. At a therapeutic dosage, DEX is expected to suppress the *IL1β*, but the results obtained in this study are consistent with a previous study highlighting that repeated injections of DEX indeed upregulated the expression of selected cytokines including *IL1β* at the transcriptional level in ileal tissues [31]. In the current experiment, birds injected with DEX had a higher intestinal permeability (elevated FITC-d in serum) in all dietary treatments but showed a complex interaction for the gene expression of *IL1β* in birds fed different diets. All amino acid supplements were able to restrain the overexpression of *IL1β* compared to control birds, but the extent of response was more pronounced for Arg and Arg-Gln fed birds.

In general, *IL-10* is produced by activated macrophages and is an anti-inflammatory cytokine with a higher expression that enhances the intestinal barrier function [32]. Although *IL-10* can be expected to be inhibited by therapeutic doses of GC, excessive or repeated injections of DEX can be detrimental, leading to upregulation of *IL-10* [33], which concurs with the results of our study. Besides, *IL-10* may have immunostimulatory properties on the production of *IFN-γ*, which can counteract its anti-inflammatory properties [34]. The overexpression of *IL-10*, *IFN-γ*, and *IL-1β* in birds injected with DEX in the current study may therefore suggest that their transcriptional expression was affected by the concentration of one or another despite their different functions. Both Arg and Gln were shown to reduce proinflammatory cytokines. For Arg, the signaling pathways include *NF-κB*, *iNOS*, and MAPK, while for Gln, *MAPK/ERK*, *mTOR*, and *NF-κB* are involved in reducing the intestinal inflammation [35]. Similarly, Thr can reduce intestinal inflammation by increasing MUC-2, IgA, and barrier function via *NF-κB*, *mTOR*, and *MAPK* [35]. We studied *NF-κB*, *mTOR*, and *MUC-2*, which all remained unaffected by the experimental treatments. This suggests that other mechanisms may have been involved in the response to the tested amino acid combinations for affecting expression of *IL1β* as well as changes in the intestinal permeability. Whole RNA sequencing may provide insights into possible mechanistic explanations in future studies. 

Apoptosis is the programmed process of death in various cells including intestinal epithelial cells. When apoptotic pathways are disrupted, unwanted, damaged or infected cells cannot be fully eliminated. In this study, two apoptotic regulating proteins of *BCL-2* and *Caspase-3* were studied by mRNA expression. These two genes were not influenced in this study by either DEX or the tested supplements. Glucocorticoids are expected to promote apoptosis and reduce cell proliferation as they increase oxidative stress in various cells [36]. The lack of effect in this study may be related to sampling time or involvement of other pathways not specifically investigated here. Various amino acids [37] and grape extracts are also shown to affect pathways of apoptosis but none of the treatments affected the selected apoptotic-related genes studied here. 

*mTOR* Complex 1 pathways are known to be affected by dietary amino acids that in turn affect protein syntheses. In this study, three genes related to this pathway including *mTOR*, *Raptor*, and *RPS6KB1* were studied. Only *Raptor* was upregulated in birds fed MIX, which had a combination of Arg, Thr, Glu, and grape extract only compared with birds fed a combination of Arg-Glu. The upregulation of *Raptor* is shown to promote protein synthesis and it interacts with *mTOR* to form a nutrient sensitive complex [38]. Therefore, it is possible that the addition of Thr and grape extract may have had some positive stimulatory impacts on *mTORC-1* through activation of *Raptor*. Given the nature of the product tested in this study, it is not possible to pinpoint the effect to a particular amino acid, but this positive impact is consistent with improved feed efficiency observed in this group of birds even though a lower concentration of amino acids were fed compared with other treatments. The positive effect of Arg on gene expression of *Raptor* and *RPS6KB1* is shown by other researchers [5]. 

Glucose absorption through the intestinal epithelium is facilitated by *SGLT1* that are apically located and play an important role in making the glucose available for maintaining cellular and organic functions. While an increase in *SGLT-1* can be perceived positive for enhancing growth performance and gut development [39], its overexpression under stress conditions may increase paracellular permeability [40] and promote fat deposition [39]. As there was no significant change in intestinal permeability in birds fed a diet supplemented with MIX, the increased expression of *SGLT-1* may have been a possible result of a synergistic stimulatory effect of grape extract and a combination of tested amino acids, which deserves further investigation. Apart from transferring peptides, *PepT-1* can be upregulated in a cell survival mechanism under stress conditions [41]. The lack of changes in expression of *PepT-1* in the current experiment was somewhat unexpected as both lower feed intake [42] and the stress stimulated by DEX [12] are shown to upregulate expression of *PepT-1*. 

Under various stress conditions including physiological stress, heat shock proteins such as *HSP70* are expected to be highly expressed as an endogenous protection mechanism to maintain cellular hemostasis and prevent apoptotic processes [43]. Noteworthy, the measurement of this gene was unaltered in the current study by either diet or DEX challenge. However, as an enzyme involved in synthesis of glutathione, *GSS* was affected independently by both diet and DEX. The response to DEX by upregulation of *GSS* may indicate an oxidative/antioxidative balance response under physiological stress induced by GC. In future studies, measurement glutathione content may provide more insight into the potential interactive effect of treatments under stress stimulated with DEX. Among tested treatments, MIX containing grape extract caused the highest expression of *GSS*, which may be related to flavonoids present in that treatment as dietary grape proanthocyanidins are shown to improve antioxidant indices such as glutathione content [44]. 

## 5. Conclusions

The present studies confirm the beneficial effect of additional Arg for improving growth performance and reducing intestinal inflammation and permeability in broiler chickens, particularly when reduced protein diets are adapted. A combination of Arg and Gln and a supplement including Arg, Gln, Thr, and grape extract was equally effective at least for the performance and reducing intestinal inflammation. While no detectable difference was observed for selected tight junction proteins, the most notable results were obtained by a significant interaction between diet and DEX for genes including *Nrf2* and *IL1β* through which tested amino acids were able to retrain the intestinal inflammation caused by stress stimulated by DEX injections. The positive effects of the tested amino acid-based solution containing grape extract at a lower inclusion rate underscore the potential for developing specifically tailored combinations of amino acids to strengthen intestinal barrier function as a new frontier to prevent or counteract stress and enteric-related issues in broiler chickens. Such approach is facilitated by understanding the mechanistic and physiological pathways that amino acids and selected compounds such as grape extract may influence. 

## Figures and Tables

**Table 1:** Composition of basal experimental grower and finisher diets.

Ingredients.	Grower	Finisher
Corn	30.000	30.000
Wheat	35.660	38.555
Soybean meal	24.959	21.513
Canola oil	3.529	4.634
Limestone	1.066	0.989
Dicalcium phosphate	1.755	1.552
Xylanase	0.005	0.005
Sodium chloride	0.141	0.156
Sodium bicarbonate	0.402	0.384
Sand	0.500	0.500
Vitamin and mineral premix ^1^	0.200	0.200
Choline Cl 70%	0.087	0.096
L-lysine HCl 78.4	0.442	0.390
DL-methionine	0.336	0.309
L-threonine	0.208	0.169
Glycine	0.232	0.215
L-Arginine	0.161	0.144
L-Valine	0.192	0.089
L-Isoleucine	0.126	0.103
Nutrient (% unless specified)		
Dry matter	90.65	90.69
AMEn (Kcal/kg)	3100	3200
Crude protein	19.50	18.00
Crude fat	5.93	7.01
Crude fiber	2.20	2.13
Digestible Arg	1.21	1.10
Digestible Lys	1.15	1.03
Digestible Met	0.58	0.54
Digestible Cys	0.27	0.26
Digestible Met+Cys	0.85	0.80
Digestible Trp	0.21	0.19
Digestible His	0.43	0.40
Digestible Phe	0.79	0.73
Digestible Leu	1.25	1.16
Digestible Ile	0.78	0.71
Digestible Thr	0.77	0.69
Digestible Val	0.93	0.78
Digestible Gly	0.86	0.80
Digestible Ser	0.76	0.71
Digestible Phe + Tyr	1.29	1.19
Digestible Glyeq	1.40	1.30
Calcium	0.87	0.79
Available phosphorus	0.44	0.40
Sodium	0.20	0.20
Potassium	0.80	0.74
Chloride	0.23	0.23

^1^ Vitamin and mineral premix provided per kilogram of diet: retinol, 12,000 IU; cholecalciferol, 5000 IU; tocopheryl acetate, 75 mg; menadione, 3 mg; thiamine, 3 mg; riboflavin, 8 mg; niacin, 55 mg; pantothenate, 13 mg; pyridoxine, 5 mg; folate, 2 mg; cyanocobalamin, 16 μg; biotin, 200 μg; cereal-based carrier, 149 mg; mineral oil, 2.5 mg; Cu (sulfate), 16 mg; Fe (sulfate), 40 mg; I (iodide), 1.25 mg; Se (selenate), 0.3 mg; Mn (sulfate and oxide), 120 mg; Zn (sulfate and oxide), 100 mg; mineral oil, 3.75 mg.

**Table 2:** Primer sequences of genes used in PCR assays.

Genes	Primer 5′–3′	Reference
Reference genes		
*GAPDH*	F: CAACCCCCAATGTCTCTGTT	[16]
	R: TCAGCAGCAGCCTTCACTAC	
*TBP*	F: GTCCACGGTGAATCTTGGTT	[16]
	R: GCGCAGTAGTACGTGGTTCTC	
Tight junction proteins		
*ZO1*	F: CCGCAGTCGTTCACGATCT	[17]
	R: GGAGAATGTCTGGAATGGTCTGA	
*ZO2*	F: GCCCAGCAGATGGATTACTT	[16]
	R: TGGCCACTTTTCCACTTTTC	
*Claudin 1*	F: AAGGTGTACGACTCGCTGCT	[16]
	R: CAGCAACAAACACACCAACC	
*Claudin 3*	F: GCCAAGATCACCATCGTCTC	[16]
	R: CACCAGCGGGTTGTAGAAAT	
Immune related and pathways		
*MUC-2*	F: CAGCACCAACTTCTCAGTTC	[5]
	R: TCTGCAGCCACACATTCTTT	
*IFN-γ*	F: ACACTGACAAGTCAAAGCCGC	[18]
	R: AGTCGTTCATCGGGAGCTTG	
*IL-6*	F: AGGACGAGATGTGCAAGAAGTTC	[19]
	R: TTGGGCAGGTTGAGGTTGTT	
*NFκB*	F: GAAGGAATCGTACCGGGAACA	[19]
	R: CTCAGAGGGCCTTGTGACAGTAA	
*Nrf2*	F: GGAAGAAGGTGCTTTTCGGAGC	[19]
	R: GGGCAAGGCAGATCTCTTCCAA	
*IL-1β*	F: CAGCCCGTGGGCATCA	[17]
	R: CTTAGCTTGTAGGTGGCGATGTT	
*IL-10*	F: ATGAACTTAACATCCAACTGCTC	[20]
	R: TGTTGCCCAGGTCGCCCAT	
Oxidative stress related		
*GSS*	F: GTACTCACTGGATGTGGGTGAAGA	[21]
	R: CGGCTCGATCTTGTCCATCAG	
*HSP-70*	F: AGCGTAACACCACCATTCC	[22]
	R: TGGCTCCCACCCTATCTC	
Protein synthesis/apoptosis		
*mTOR*	F: CATGCAATGATGGAGCGTGG	[5]
	R: GCAGCTGCTTTGAGATACGC	
*Raptor*	F: GCTGAGACCGCTTCTTGTCT	[5]
	R: GTTCAGCTGGCATGTACGGA	
*RPS6KB1*	F: TGGAAGCCATGGGCTCAAAT	[5]
	R: GTACAGCCACACCTCCTGAC	
*Caspase-3*	F: CAGCTGAAGGCTCCTGGTTT	[20]
	R: GCCACTCTGCGATTTACACG	
*Bcl-2*	F: GATGACCGAGTACCTGAACC	[20]
	R: CAGGAGAAATCGAACAAAGGC	
Transporters related to gut health		
*SGLT-1*	F: TGCCGGAGTATCTGAGGAAG	[16]
	R: CCCCATGGCCAACTGTATAA	
*PepT-1*	F: ACACGTTTGTTGCTCTGTGC	[16]
	R: GACTGCCTGCCCAATTGTAT	

*GAPDH*: Glyceraldehyde-3-phosphate dehydrogenase; *TBP*: TATA-box binding protein; *ZO1*: Zonula occludens 1; *ZO2*: Zonula occludens 2; *MUC-2*: Mucin 2; *IFN-γ*: Interferon gamma; *IL-6*: Interleukin 6; *NFκB*: Nuclear factor kappa B; *Nrf2*: Nuclear factor-erythroid factor 2-related factor 2; *IL-1β*: Interleukin-1β; *IL-10*: Interleukin-10; *GSS*: Glutathione synthetase; *HSP-70*: Heat shock protein 70; *mTOR*: Mammalian target of rapamycin; *Raptor*: Regulatory-associated protein of *mTOR*; *RPS6KB1*: Ribosomal protein S6 kinase beta-1; *Bcl-2*: B-cell lymphoma 2; *SGLT-1*: Sodium/glucose cotransporter 1; *PepT-1*: Peptide transporter 1.

**Table 3:** Body weight (BW, g/bird) and body weight gain (BWG, g/bird) of broiler chickens fed experimental diets (experiment 1).

Treatments	BW 7	BW 21	BW 35	BWG (d 7–21)	BWG (d 21–35)	BWG (d 7–35)
Control (CON)	174	1064	2454	890	1391	2280
CON + Arg	167	1111	2515	944	1405	2348
CON + Arg + Gln	176	1106	2518	931	1412	2343
CON + MIX ^1^	174	1111	2556	937	1446	2383
SEM ^2^	1.6	10.5	18.7	9.4	10.8	17.5
*p* value	0.305	0.333	0.309	0.203	0.346	0.249

^1^ MIX contained Arg, Gln, Thr, and grape extract. ^2^ Pooled standard error of the mean (*n* = 24).

**Table 4:** Feed intake (FI, g/bird) and feed conversion ratio (FCR, g feed/g weight gain) of broiler chickens fed experimental diets (experiment 1).

Treatments	FI (d 7–21)	FI (d 21–35)	FI (d 7–35)	FCR (d 7–21)	FCR (d 21–35)	FCR (d 7–35)
Control (CON)	1128	1980	3108	1.270 ^a^	1.423	1.363 ^a^
CON + Arg	1145	1953	3097	1.214 ^b^	1.391	1.319 ^b^
CON + Arg + Gln	1132	1978	3110	1.217 ^b^	1.401	1.328 ^b^
CON + MIX ^1^	1133	2040	3173	1.209 ^b^	1.411	1.331 ^b^
SEM ^2^	9.6	14.8	22.5	0.0061	0.0048	0.0031
*p* value	0.937	0.223	0.633	0.006	0.142	0.0004

^a,b^ Means not sharing a same superscript differ significantly at the *p* level shown (*n* = 24). ^1^ MIX contained Arg, Gln, Thr, and grape extract. ^2^ Pooled standard error of the mean.

**Table 5:** Indicative 1-week (day 14 to 21 of age) performance of individually housed birds subjected to dexamethasone (DEX) or sham injections (experiment 2).

Main Effects	Weight Gain (g/bird)	Feed Intake (g/bird)	Feed Conversion Ratio
Diet			
Control (CON)	381	591	1.672
CON + Arg	382	583	1.634
CON + Arg + Gln	382	586	1.672
CON + MIX ^1^	385	595	1.668
Challenge			
Sham	490 ^a^	622 ^a^	1.271 ^a^
DEX	274 ^b^	556 ^b^	2.052 ^b^
SEM ^2^	4.8	5.4	0.0164
Source of variation			
DEX	<0.0001	<0.0001	<0.0001
Diet	0.989	0.872	0.805
Diet × DEX	0.653	0.545	0.880

^a,b^ Means sharing a different superscript differ significantly at the *p* level shown. ^1^ MIX contained Arg, Gln, Thr and grape extract. ^2^ Pooled standard error of the mean (*n* = 96).

**Table 6:** Relative weights (g/100 g BW) of liver, spleen, and bursa of broiler chickens fed four experimental diets subjected to dexamethasone (DEX) or sham injections (experiment 2).

Main Effects	Liver	Spleen	Bursa
Diet			
Control (CON)	3.67 ^a^	0.062	0.156
CON + Arg	3.25 ^b^	0.055	0.148
CON + Arg + Gln	3.42 ^b^	0.063	0.155
CON + MIX ^1^	3.41 ^b^	0.057	0.168
Challenge			
Sham	2.65 ^b^	0.074 ^a^	0.233 ^a^
DEX	4.23 ^a^	0.044 ^b^	0.078 ^b^
SEM ^2^	0.035	0.0012	0.0034
Source of variation			
DEX	<0.0001	<0.0001	<0.0001
Diet	0.0009	0.122	0.498
Diet × DEX	0.688	0.532	0.167

^a,b^ Means sharing a different superscript differ significantly at the *p* level shown. ^1^ MIX contained Arg, Gln, Thr, and grape extract. ^2^ Pooled standard error of the mean (*n* = 96).

**Table 7:** Relative gene expression of tight junction proteins in ileum of broilers (day 21) subjected to dexamethasone (DEX) or sham injections (experiment 2).

Treatments		*Claudin 1*	*Claudin 3*	*ZO1*	*ZO2*
Control (CON)		0.614	0.630	1.192	0.673
Control (CON)	DEX	0.792	1.075	1.124	1.042
CON + Arg		0.782	0.830	1.259	0.832
CON + Arg	DEX	0.589	1.041	1.052	0.954
CON + Arg + Gln		0.581	0.729	1.136	0.739
CON + Arg + Gln	DEX	0.847	1.040	1.070	0.905
CON + MIX ^1^		0.811	0.836	1.347	0.709
CON + MIX	DEX	0.584	0.973	1.245	0.967
SEM ^2^		0.0382	0.0302	0.0306	0.0219
Main effects					
Sham		0.697	0.756 ^b^	1.233	0.738 ^b^
DEX		0.703	1.032 ^a^	1.122	0.967 ^a^
Control (CON)		0.703	0.852	1.158	0.857
CON + Arg		0.685	0.935	1.155	0.893
CON + Arg + Gln		0.714	0.884	1.103	0.821
CON + MIX		0.697	0.904	1.296	0.838
*p* value					
DEX		0.935	<0.0001	0.079	<0.0001
Diet		0.994	0.801	0.158	0.694
DEX × Diet		0.056	0.317	0.833	0.215

^a,b^ Means sharing a different superscript differ significantly at the *p* level shown. ^1^ MIX contained Arg, Gln, Thr, and grape extract. ^2^ Pooled standard error of the mean (*n* = 48).

**Table 8:** Relative gene expression of MUC-2, immune-related mechanistic genes and mTOR in ileum of broilers (day 21) subjected to dexamethasone (DEX) or sham injections (experiment 2).

Treatments		*MUC2*	*IFNy*	*NFκB*	*Nrf2*	*IL1β*	*IL6*	*IL10*	*mTOR*
Control (CON)		1.072	0.975	1.363	1.837 ^c^	0.475 ^bc^	0.59	0.404	0.838
Control (CON)	DEX	1.254	3.346	1.760	2.539 ^a^	1.234 ^a^	0.69	1.184	0.934
CON + Arg		1.028	1.291	1.480	2.158 ^abc^	0.518 ^bc^	0.60	0.389	0.868
CON + Arg	DEX	1.249	2.337	1.624	1.976 ^bc^	0.730 ^bc^	0.43	0.975	0.896
CON + Arg + Gln		1.179	0.814	1.561	2.036 ^abc^	0.473 ^bc^	0.61	0.397	1.007
CON + Arg + Gln	DEX	1.113	2.159	1.599	2.371 ^ab^	0.472 ^bc^	0.60	0.919	0.907
CON + MIX ^1^		1.093	1.230	1.535	2.305 ^abc^	0.436 ^c^	0.76	0.583	1.033
CON + MIX	DEX	1.291	1.065	1.515	2.032 ^abc^	0.831 ^b^	0.66	0.871	0.928
SEM ^2^		0.0408	0.2151	0.0405	0.0654	0.0466	0.0404	0.0439	0.0257
Main effects									
Sham		1.093	1.078 ^b^	1.485	2.084	0.475	0.638	0.443 ^b^	0.936
DEX		1.226	2.227 ^a^	1.624	2.229	0.817	0.593	0.987 ^a^	0.916
Control (CON)		1.163	2.160	1.561	2.188	0.854	0.638	0.794	0.885
CON + Arg		1.138	1.814	1.552	2.067	0.624	0.514	0.682	0.882
CON + Arg + Gln		1.146	1.486	1.579	2.203	0.472	0.604	0.657	0.957
CON + MIX		1.192	1.147	1.525	2.168	0.633	0.707	0.726	0.980
*p* value									
DEX		0.109	0.0109	0.093	0.272	0.0007	0.5797	<0.0001	0.698
Diet		0.968	0.394	0.970	0.881	0.049	0.405	0.707	0.428
DEX × Diet		0.573	0.237	0.289	0.039	0.042	0.678	0.278	0.438

^a–c^ Means within the same column with different superscript differ significantly for the *p* level shown for main effects and *p* < 0.05 for treatment effect when there is a significant DEX #xD7; Diet interaction. ^1^ MIX contained Arg, Gln, Thr, and grape extract. ^2^ Pooled standard error of the mean (*n* = 48).

**Table 9:** Relative gene expression of genes related to protein synthesis, apoptosis, nutrient transporters, and oxidative stress in ileum of broilers (day 21) subjected to dexamethasone (DEX) or sham injections (experiment 2).

Treatments		*Bcl2*	*Caspase-3*	*Raptor*	*RPS6KB1*	*Pept1*	*SGLT1*	*GSS*	*HSP70*
Control (CON)		0.547	0.900	0.319	2.032	0.235	0.225	0.506	0.983
Control (CON)	DEX	0.558	1.011	0.354	2.176	0.196	0.233	0.629	0.856
CON + Arg		0.577	0.929	0.340	2.224	0.248	0.263	0.541	0.939
CON + Arg	DEX	0.461	1.144	0.333	2.140	0.219	0.273	0.648	0.715
CON + Arg + Gln		0.545	0.936	0.337	2.075	0.232	0.262	0.549	0.851
CON + Arg + Gln	DEX	0.555	1.096	0.280	2.213	0.278	0.256	0.534	0.838
CON + MIX ^1^		0.609	1.125	0.396	2.467	0.251	0.308	0.611	0.984
CON + MIX	DEX	0.506	1.148	0.368	2.395	0.359	0.486	0.708	0.833
SEM ^2^		0.0191	0.0341	0.0090	0.0544	0.0155	0.0199	0.0149	0.0364
Main effects									
Sham		0.569	0.972	0.348	2.199	0.241	0.264	0.551 ^b^	0.939
DEX		0.519	1.099	0.333	2.230	0.263	0.312	0.629 ^a^	0.810
Control (CON)		0.552	0.955	0.336 ^ab^	2.104	0.215	0.229 ^b^	0.567 ^b^	0.919
CON + Arg		0.518	1.036	0.336 ^ab^	2.182	0.233	0.268 ^b^	0.594 ^ab^	0.826
CON + Arg + Gln		0.549	1.016	0.308 ^b^	2.144	0.255	0.258 ^b^	0.541 ^b^	0.844
CON + MIX		0.557	1.136	0.381 ^a^	2.431	0.305	0.396 ^a^	0.659 ^a^	0.908
*p* value									
DEX		0.203	0.069	0.432	0.774	0.487	0.240	0.012	0.084
Diet		0.888	0.316	0.049	0.156	0.213	0.025	0.047	0.756
DEX × Diet		0.483	0.787	0.350	0.797	0.307	0.324	0.354	0.784

^a,b^ Means within the same column with different superscript differ significantly for the *p* level shown for main effects and *p* < 0.05 for treatment effect when there is a significant DEX × Diet interaction. ^1^ MIX contained Arg, Gln, Thr, and grape extract. ^2^ Pooled standard error of the mean (*n* = 48).

## Data Availability

The data presented in this study are available on request from the corresponding author.

## Supplementary Materials

The following are available online at https://www.mdpi.com/article/10.3390/ani11082416/s1, Table S1: Amino acid analysis of experimental diets.

## References

[1] Bortoluzzi C., Rochell S., Applegate T. (2017). Threonine, arginine, and glutamine: Influences on intestinal physiology, immunology, and microbiology in broilers. Poult. Sci..

[2] Chalvon-Demersay T., Luise D., Le Floc’h N., Tesseraud S., Lambert W., Bosi P., Trevisi P., Beaumont M., Corrent E. (2021). Functional Amino Acids in Pigs and Chickens: Implication for Gut Health. Front. Vet. Sci..

[3] Wu G., Knabe D.A. (1995). Arginine synthesis in enterocytes of neonatal pigs. Am. J. Physiol. Regul. Integr. Comp. Physiol..

[4] Jacobi S.K., Odle J. (2012). Nutritional factors influencing intestinal health of the neonate. Adv. Nutr. Int. Rev. J..

[5] Tan J., Applegate T.J., Liu S., Guo Y., Eicher S.D. (2014). Supplemental dietary L-arginine attenuates intestinal mucosal disruption during a coccidial vaccine challenge in broiler chickens. Br. J. Nutr..

[6] Zhang B., Lv Z., Li H., Guo S., Liu D., Guo Y. (2017). Dietary l-arginine inhibits intestinal Clostridium perfringens colonisation and attenuates intestinal mucosal injury in broiler chickens. Br. J. Nutr..

[7] Barekatain R., Chrystal P., Howarth G., McLaughlan C., Gilani S., Nattrass G. (2019). Performance, intestinal permeability, and gene expression of selected tight junction proteins in broiler chickens fed reduced protein diets supplemented with arginine, glutamine, and glycine subjected to a leaky gut model. Poult. Sci..

[8] Zhang Q., Chen X., Eicher S.D., Ajuwon K.M., Applegate T.J. (2016). Effect of threonine deficiency on intestinal integrity and immune response to feed withdrawal combined with coccidial vaccine challenge in broiler chicks. Br. J. Nutr..

[9] Gilani S., Howarth G.S., Tran C.D., Kitessa S.M., Forder R.E., Barekatain R., Hughes R.J. (2018). Effects of delayed feeding, sodium butyrate and glutamine on intestinal permeability in newly-hatched broiler chickens. J. Appl. Anim. Res..

[10] Pandey K.B., Rizvi S.I. (2009). Plant polyphenols as dietary antioxidants in human health and disease. Oxidative Med. Cell. Longev..

[11] Gilani S., Chrystal P.V., Barekatain R. (2021). Current experimental models, assessment and dietary modulations of intestinal permeability in broiler chickens. Anim. Nutr..

[12] Barekatain R., Nattrass G., Tilbrook A., Chousalkar K., Gilani S. (2019). Reduced protein diet and amino acid concentration alter intestinal barrier function and performance of broiler chickens with or without synthetic glucocorticoid. Poult. Sci..

[13] Bouvet R., Alleno C., Chalvon-Demersay T., Lambert W. (2019). Effect of seven different feed programs on broiler performances, ZT1904 experiment.

[14] Wideman R., Pevzner I. (2012). Dexamethasone triggers lameness associated with necrosis of the proximal tibial head and proximal femoral head in broilers. Poult. Sci..

[15] Vicuña E., Kuttappan V., Galarza-Seeber R., Latorre J., Faulkner O., Hargis B., Tellez G., Bielke L. (2015). Effect of dexamethasone in feed on intestinal permeability, differential white blood cell counts, and immune organs in broiler chicks. Poult. Sci..

[16] Gilani S., Howarth G., Nattrass G., Kitessa S., Barekatain R., Forder R., Tran C., Hughes R. (2018). Gene expression and morphological changes in the intestinal mucosa associated with increased permeability induced by short-term fasting in chickens. J. Anim. Physiol. Anim. Nutr..

[17] Chen J., Tellez G., Richards J.D., Escobar J. (2015). Identification of potential biomarkers for gut barrier failure in broiler chickens. Front. Vet. Sci..

[18] Brisbin J.T., Gong J., Parvizi P., Sharif S. (2010). Effects of lactobacilli on cytokine expression by chicken spleen and cecal tonsil cells. Clin. Vaccine Immunol..

[19] Lee M., Lin W., Wang S., Lin L., Yu B., Lee T. (2018). Evaluation of potential antioxidant and anti-inflammatory effects of Antrodia cinnamomea powder and the underlying molecular mechanisms via Nrf2-and NF-κB-dominated pathways in broiler chickens. Poult. Sci..

[20] Xiao M., Mi Y., Liu L., Lv C., Zeng W., Zhang C., Li J. (2018). Taurine regulates mucosal barrier function to alleviate lipopolysaccharide-induced duodenal inflammation in chicken. Amino Acids.

[21] Balogh K., Kövesi B., Zándoki E., Kulcsár S., Ancsin Z., Erdélyi M., Dobolyi C., Bata-Vidács I., Inotai K., Szekeres A. (2019). Effect of sterigmatocystin or aflatoxin contaminated feed on lipid peroxidation and glutathione redox system and expression of glutathione redox system regulatory genes in broiler chicken. Antioxidants.

[22] Yi D., Hou Y., Tan L., Liao M., Xie J., Wang L., Ding B., Yang Y., Gong J. (2016). N-acetylcysteine improves the growth performance and intestinal function in the heat-stressed broilers. Anim. Feed Sci. Technol..

[23] Aviagen (2019). Ross 308 Broiler: Performance Objectives, 2019.

[24] Barekatain R., Toghyani M. (2019). High dietary zinc and glutamine do not improve the performance or reduce excreta moisture of broiler chickens fed diets with and without magnesium supplementation. Poult. Sci..

[25] Bartell S., Batal A. (2007). The effect of supplemental glutamine on growth performance, development of the gastrointestinal tract, and humoral immune response of broilers. Poult. Sci..

[26] Abdulkarimi R., Shahir M.H., Daneshyar M. (2019). Effects of dietary glutamine and arginine supplementation on performance, intestinal morphology and ascites mortality in broiler chickens reared under cold environment. Asian-Australas. J. Anim. Sci..

[27] Yin G., Cao L., Du J., Jia R., Kitazawa T., Kubota A., Teraoka H. (2017). Dexamethasone-induced hepatomegaly and steatosis in larval zebrafish. J. Toxicol. Sci..

[28] Chelakkot C., Ghim J., Ryu S.H. (2018). Mechanisms regulating intestinal barrier integrity and its pathological implications. Exp. Mol. Med..

[29] Wu M., Xiao H., Shao F., Tan B., Hu S. (2020). Arginine accelerates intestinal health through cytokines and intestinal microbiota. Int. Immunopharmacol..

[30] Rawat M., Nighot M., Al-Sadi R., Gupta Y., Viszwapriya D., Yochum G., Koltun W., Ma T.Y. (2020). IL1B Increases intestinal tight junction permeability by up-regulation of MIR200C-3p, which degrades occludin mRNA. Gastroenterology.

[31] Barekatain R., Chrystal P.V., Gilani S., McLaughlan C.J. (2020). Expression of selected genes encoding mechanistic pathways, nutrient and amino acid transporters in jejunum and ileum of broiler chickens fed a reduced protein diet supplemented with arginine, glutamine and glycine under stress stimulated by dexamethasone. J. Anim. Physiol. Anim. Nutr..

[32] Lorén V., Cabré E., Ojanguren I., Domènech E., Pedrosa E., García-Jaraquemada A., Mañosa M., Manyé J. (2015). Interleukin-10 enhances the intestinal epithelial barrier in the presence of corticosteroids through p38 MAPK activity in Caco-2 monolayers: A possible mechanism for steroid responsiveness in ulcerative colitis. PLoS ONE.

[33] Zeng S., Qiao H., Lv X.-W., Fan D., Liu T., Xie D. (2017). High-dose dexamethasone induced LPS-stimulated rat alveolar macrophages apoptosis. Drug Des. Dev. Ther..

[34] Herfarth H., Schölmerich J. (2002). IL-10 therapy in Crohn’s disease: At the crossroads. Gut.

[35] He F., Wu C., Li P., Li N., Zhang D., Zhu Q., Ren W., Peng Y. (2018). Functions and signaling pathways of amino acids in intestinal inflammation. BioMed Res. Int..

[36] Ranta F., Avram D., Berchtold S., Düfer M., Drews G., Lang F., Ullrich S. (2006). Dexamethasone induces cell death in insulin-secreting cells, an effect reversed by exendin-4. Diabetes.

[37] Li P., Yin Y.-L., Li D., Kim S.W., Wu G. (2007). Amino acids and immune function. Br. J. Nutr..

[38] Kim D.-H., Sarbassov D.D., Ali S.M., King J.E., Latek R.R., Erdjument-Bromage H., Tempst P., Sabatini D.M. (2002). mTOR interacts with raptor to form a nutrient-sensitive complex that signals to the cell growth machinery. Cell.

[39] Shibata M., Takahashi T., Kozakai T., Kakudo M., Kasuga S., Azuma Y., Kurose Y. (2019). Active transport of glucose across the jejunal epithelium decreases with age in broiler chickens. Poult. Sci..

[40] de Punder K., Pruimboom L. (2015). Stress induces endotoxemia and low-grade inflammation by increasing barrier permeability. Front. Immunol..

[41] Chen H., Pan Y., Wong E.A., Webb K.E. (2005). Dietary protein level and stage of development affect expression of an intestinal peptide transporter (cPepT1) in chickens. J. Nutr..

[42] Ihara T., Tsujikawa T., Fujiyama Y., Bamba T. (2000). Regulation of PepT1 peptide transporter expression in the rat small intestine under malnourished conditions. Digestion.

[43] Shehata A.M., Saadeldin I.M., Tukur H.A., Habashy W.S. (2020). Modulation of heat-shock proteins mediates chicken cell survival against thermal stress. Animals.

[44] Han M., Song P., Huang C., Rezaei A., Farrar S., Brown M.A., Ma X. (2016). Dietary grape seed proanthocyanidins (GSPs) improve weaned intestinal microbiota and mucosal barrier using a piglet model. Oncotarget.

