# Supplementary material for: Intestinal Barrier Function and Performance of Broiler Chickens Fed Additional Arginine, Combination of Arginine and Glutamine or an Amino Acid-Based Solution
Source: Animals (Basel). 2021 Aug 17;11(8):2416. doi: 10.3390/ani11082416 (PMC8388668; doi:10.3390/ani11082416)
Supplement: Supplementary file 1 [file animals-11-02416-s001.zip › animals-1346911-supplementary.pdf]

Table S1. Analyzed total amino acid content of grower and finisher experimental diets (g/kg) [1].

	G1	G2	G3	G4	F1	F2	F3	F4
His	4.9	4.8	4.8	4.8	4.5	4.4	4.6	4.6
Ser	9.3	9.3	9.3	9.2	8.7	8.5	8.7	8.7
Arg	12.8	17	15.1	13	11.8	15.6	14.1	12.4
Gly	10.1	10	10.1	10	9.4	9.2	9.5	9.6
Asp	17.4	17.1	17.2	17.1	15.9	15.4	16	15.9
Glutamic acid	39.7	39.4	41.7	39.4	38	37.3	40.4	38.2
Thr	8.8	8.8	8.8	8.9	8	7.7	8	8.3
Ala	8.5	8.4	8.4	8.5	8	7.8	8	8
Pro	12.6	12.5	12.5	12.5	12.2	12.1	12.1	12.2
Lys	12.8	12.5	12.6	12.3	11.4	11.1	12.1	11.5
Tyr	4.7	4.6	4.7	4.6	4.5	4.1	4.4	4.5
Met	4.7	5	5.6	5.5	4.8	4.6	4.5	4.7
Val	10.6	10.5	10.5	10.3	9.1	8.9	9.2	9.2
Ile	9.2	9.1	9.1	8.9	8.4	8.2	8.4	8.4
Leu	15.5	15.4	15.4	15.4	14.6	14.3	14.6	14.6
Phe	9.4	9.3	9.3	9.2	8.8	8.6	8.8	8.8

[1] Each value represents average of duplicate analysis; Grower (G); G1: control; G2: Control + Arg; G3: Control + Arg + Gln; G4: Control + MIX; Finisher diets (F); F1: control; F2: Control + Arg; F3: Control + Arg + Gln; F4: Control + MIX .
